# Impacts of Elevated Atmospheric CO_2_ and O_3_ on Paper Birch (*Betula papyrifera*): Reproductive Fitness

**DOI:** 10.1100/tsw.2007.42

**Published:** 2007-03-21

**Authors:** Joseph N. T. Darbah, Mark E. Kubiske, Neil Nelson, Elina Oksanen, Elina Vaapavuori, David F. Karnosky

**Affiliations:** ^1^ Michigan Technological University, Houghton, MI, USA; ^2^ USDA Forest Service, North Central Research Station, Rhinelander, WI, USA; ^3^ University of Joensuu, Kuopio, Finland; ^4^ Finnish Forest Research Institute, Suonenjoki, Finland

**Keywords:** seed production and germination, paper birch, flowering, elevated CO_2_ and O

## Abstract

Atmospheric CO_2_ and tropospheric O_3_ are rising in many regions of the world. Little is known about how these two commonly co-occurring gases will affect reproductive fitness of important forest tree species. Here, we report on the long-term effects of CO_3_ and O_3_ for paper birch seedlings exposed for nearly their entire life history at the Aspen FACE (Free Air Carbon Dioxide Enrichment) site in Rhinelander, WI. Elevated CO_2 _increased both male and female flower production, while elevated O_3_ increased female flower production compared to trees in control rings. Interestingly, very little flowering has yet occurred in combined treatment. Elevated CO_2_ had significant positive effect on birch catkin size, weight, and germination success rate (elevated CO_2_ increased germination rate of birch by 110% compared to ambient CO_2_ concentrations, decreased seedling mortality by 73%, increased seed weight by 17%, increased root length by 59%, and root-to-shoot ratio was significantly decreased, all at 3 weeks after germination), while the opposite was true of elevated O_3_ (elevated O_3_ decreased the germination rate of birch by 62%, decreased seed weight by 25%, and increased root length by 15%). Under elevated CO_2_, plant dry mass increased by 9 and 78% at the end of 3 and 14 weeks, respectively. Also, the root and shoot lengths, as well as the biomass of the seedlings, were increased for seeds produced under elevated CO_2_, while the reverse was true for seedlings from seeds produced under the elevated O_3_. Similar trends in treatment differences were observed in seed characteristics, germination, and seedling development for seeds collected in both 2004 and 2005. Our results suggest that elevated CO_2_ and O_3_ can dramatically affect flowering, seed production, and seed quality of paper birch, affecting reproductive fitness of this species.

### INTRODUCTION

Atmospheric $CO_2$ and tropospheric $O_3$ have increased dramatically in the past century and are projected to continue to rise through this current century[1,2]. Large areas of the world's forests will be exposed to sharply elevated concentrations of these two co-occurring greenhouse gases, therefore, it is important to understand how these two gases will affect forest ecosystem structure and function. For example, very little is known about how these two gases will affect reproductive fitness of long-lived forest trees. Until recently, with the onset of long-term open-air exposure systems, it was nearly impossible to examine *in situ* effects of rising greenhouse gases on forest trees that generally have long juvenile periods.

### MATERIALS AND METHODS

#### Study Site

The experiment was carried out at the Aspen FACE (Free Air Carbon Dioxide Enrichment) facility in the U.S. state of Wisconsin. The Aspen FACE project was established in 1997 as the first open-air facility to examine the responses of forest trees to interacting $CO_2$ and $O_3$[3]. The facility is located at the USDA Forest Service, Harshaw experimental farm near Rhinelander, WI[4]. The experimental site consists of four rings each of control (ambient air; $CO_2$ concentrations of about 360 ppm), elevated $CO_2$ (target of 560 ppm), elevated $O_3$ (46.4–55.5 ppb) (1.5× ambient), and elevated $CO_2$ + elevated $O_3$ conditions in triplicate rings of 30-m diameter each[3].

#### Flowering

Data on flowering in 2004–2006 was collected by scoring the quantity of flowers on each tree in each treatment (score 0 = no flowers; score 1 = between 1 and 10 flowers; score 2 = between 10 and 100 flowers, and score 3 = more than 100 flowers) for both male and female flowers. This nonlinear scale was used due to the difficulty in assessing the canopy of individual trees in the stands to count the amount of flowers. Quantity of seed-bearing catkins produced was scored in the same way during the 2004 and 2005 growing seasons.

#### Seed Production

Birch catkins were collected from all rings where seed was produced in 2004 and 2005. The mean catkin length, diameter, and mass per 100 seeds (isolated seeds from catkins) were computed and analyzed for treatment differences.

#### Seed Germination

Healthy looking seeds from all the treatments were germinated under greenhouse conditions. One hundred seeds per treatment were sown and replicated four times, making 400 seeds per treatment for each of the two seed years (2004 and 2005).

#### Seedling Development Measurements

All seeds were monitored twice daily for radical emergence and on emergence, hypocotyl height and cotyledon length were measured as well as mean height. Height measurements were taken on a weekly basis for 4 months. Biomass of seedlings was determined by oven drying the roots and shoots at 70$^{o}$C for 48 h.

### Experimental Design

The Aspen FACE project is a full factorial experiment with three replicate 30-m diameter rings of four treatments[5]. Planting material of the Aspen FACE project was chosen to represent northern hardwood forests. Aspen, maple, and birch are the dominant species growing naturally in the northern Great Lakes hardwood forests. Seed sources for birch and maple were from northern Michigan sources and five aspen clones were selected on the basis of sensitivity to $O_3$ and $CO_2$[6]. Young seedlings of maple and birch, and rooted cuttings of aspen, were planted in FACE rings in the summer of 1997. All trees were planted in 1- × 1-m spacing. These rings were fumigated from 1998 onwards and average concentrations of the $CO_2$ and $O_3$ are described in Karnosky et al.[5,6]. Detailed information about the planting design, location, and gas exposure in the Aspen FACE study is given in Karnosky et al.[4] and Dickson et al.[3].

### Data Analysis

Analysis of variance (ANOVA) was used to test for significant differences between treatments. Least significant difference (LSD) was computed for determination of significant differences between treatments. F-test significant at $p < 0.05$ level[7] was used. Average values computed ± standard errors (SE) presented and different letters are used to indicate significant differences. The PROC GLM component of the SAS statistical software (by SAS Inst.) was used in carrying out this analysis.

## RESULTS

### Flowering

Birch trees under elevated $CO_2$ and elevated $O_3$ flowered earlier in their life cycle than trees in control or elevated $CO_2$ + elevated $O_3$ treatments (data not provided), female flower production was stimulated under elevated $O_3$ treatments, while elevated $CO_2$ stimulated male flower production. However, there was no statistically significant difference between the treatments as $p$ value was 0.09. The total number of trees that flowered increased by 139% under elevated $CO_2$ and by 40% under elevated $O_3$. No flowers were produced in the combined treatment. With respect to the quantity of flowers produced, elevated $CO_2$ had 262% increase, while that of elevated $O_3$ had 75% increase compared to the control treatment.

### Seed Production

Elevated $CO_2$ increased the mass of birch catkins by 13%, while elevated $O_3$ decreased the mass of birch catkins by 8% (Table 1). However, the differences were not significant as $p$ was greater than 0.05 at alpha level of 0.05. Birch seeds produced under elevated $CO_2$ had higher mass than control (12 and 17.4% for 2004 and 2005 seeds, respectively), while the opposite was true of seeds produced under elevated $O_3$ (22 and 25% decrease in seeds from 2004 and 2005, respectively). There were significant treatment differences between the weights of seeds produced under control, elevated $CO_2$, and $O_3$ treatments with a $p$ value of 0.01 and 0.009 for the seeds collected from the 2004 and 2005 growing season, respectively (Fig. 1).

### Germination

The effect of elevated $CO_2$ and $O_3$ on seed germination was very pronounced with significant treatment differences in both years. Seed produced under elevated $CO_2$ increased seed germination rate in each of

**TABLE 1**
**Characteristics (Mean ± SE) of Paper Birch Catkins, Seeds, and Seedlings from Seed Produced under Elevated $CO_2$ or $O_3$, Compared to that Produced in Ambient Conditions**

| Parameter | Control | Elevated $CO_2$ | Elevated $O_3$ |
|---|---|---|---|
| Catkin length (mm) | 33.04 ± 1.50 a* | 27.85 ± 0.87 a | 30.80 ± 2.35 a |
| Catkin diameter (mm) | 7.41 ± 0.20 a | 8.46 ± 0.44 a | 7.39 ± 0.38 a |
| Catkin mass (g) | 0.64 ± 0.05 a | 0.72 ± 0.07 a | 0.59 ± 0.06 a |
| Seed wt/100 (mg) (2004) | 22.90 ± 0.33 b | 25.57 ± 0.16 a | 17.89 ± 0.39 c |
| Seed wt/100 (mg) (2005) | 37.81 ± 4.97 ab | 44.38 ± 4.85 a | 28.36 ± 1.58 b |
| Germination rate (2004) | 10.50 ± 0.5 b | 18.00 ± 2.00 a | 3.00 ± 1.00 c |
| Germination rate (2005) | 20.75 ± 5.02 b | 43.50 ± 7.93 a | 8.00 ± 1.41 c |
| Seedling mortality (%) | 2.75 ± 1.11 a | 0.75 ± 0.48 a | 2.50 ± 0.96 a |
| Root length (mm) | 28.15 ± 5.56 b | 44.68 ± 5.86 a | 32.3 ± 8.31 ab |
| Shoot length (mm) | 5.50 ± 0.77 a | 6.65 ± 0.58 a | 5.1 ± 0.44 a |
| Cotyledon length (mm) | 2.64 ± 0.30 a | 2.98 ± 0.27 a | 2.5 ± 0.25 a |
| Root mass (g) | 0.72 ± 0.26 a | 1.84 ± 0.49 a | 1.55 ± 0.40 a |
| Shoot mass (g) | 1.26 ± 0.03 b | 2.09 ± 0.30 a | 1.77 ± 0.34 ab |
| Seedling mass (g) | 1.97 ± 0.31 a | 3.93 ± 0.53 a | 3.32 ± 0.78 a |
| Dry Root mass (g) | 0.30 ± 0.09 a | 0.48 ± 0.10 a | 0.40 ± 0.07 a |
| Dry Shoot mass (g) | 0.46 ± 0.09 a | 0.49 ± 0.12 a | 0.61 ± 0.10 a |
| Dry Seedling mass (g) | 0.79 ± 0.09 a | 0.86 ± 0.13 a | 1.10 ± 0.15 a |

* Means are of three replicates. Means within a row not followed by the same letter are significantly different ($p < 0.05$).

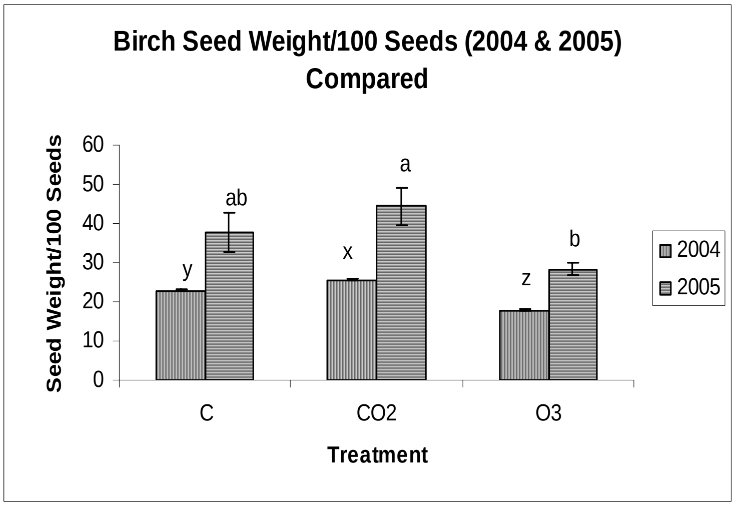

**FIGURE 1.** Weight per 100 seeds of birch collected from 2004 and 2005 growing seasons. The letters a, ab, and b; x, y, and z show significant differences between treatments in 2004 and 2005 growing seasons, respectively, at 0.05 significance level.

the 2 years: 71% in 2004 and 110% in 2005 seeds, respectively. Seed produced under elevated $O_3$ had a greatly decreased germination rate: 71% decrease in 2004 and 61% decrease in 2005 seeds, respectively, as seen from Fig. 2.

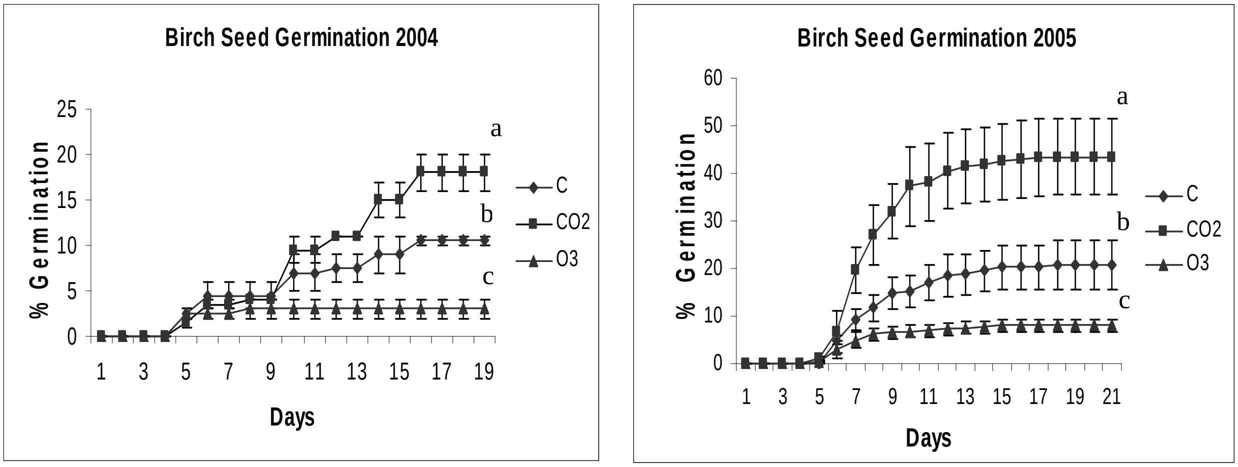

**FIGURE 2.** Germination of birch seed from trees exposed to background or elevated $CO_2$ and $O_3$; seeds from 2004 growing season (left), seeds from 2005 growing season (right). Letters a, b, and c denote significant differences in treatments at alpha level of 0.05.

### DISCUSSION

Sexual reproductive development is an important stage in the life cycle of trees, as any adverse effects on the processes involved might have significant implications for the productivity of plants and their survival[8]. Information currently available on the impacts of elevated $CO_2$ and $O_3$ on forest tree reproductive fitness is very scanty compared to agricultural crops[9]. In this study, we observed an early reproductive maturation time under elevated $CO_2$, as reported by Ladeau and Clark[10], and a similar observation under elevated $O_3$, as opposed to control and the combination treatments. This early onset of reproduction maturation may be translated into an increase in the number of pollen-producing trees (under elevated $CO_2$) and, hence, greater pollen production[10].

Elevated $CO_2$ had positive impacts on the seed weight, as seeds from this source were significantly higher in weight than control, while that from elevated $O_3$ was significantly lower in weight relative to control (Fig. 2). Hussain et al.[9], Jablonski et al.[11], and Wang[12] also reported similar increases in seed weight under elevated $CO_2$. Hussain et al.[9] reported that the increase in seed weight of seeds produced under elevated $CO_2$ was due to an increased amount of carbohydrate, lipids, and proteins, about 91% greater weight, which had profound effect on seed germination and seedling growth. This is because such seeds have stored much food for fast growth of the new seedlings. The opposite effect was observed in seeds produced under elevated $O_3$. The seeds produced under elevated $O_3$ had much less stored carbohydrate, lipids, and proteins for the newly developing seedling to depend on and, hence, the slow growth rate. $O_3$ also reduces the germination of pollen[8] and, hence, reduces the number of completely fertilized ovules for viable seed production, which ultimately results in reduction of germination.

The process of germination is an energy-demanding one that is fuelled by respiration of fats. Lipid reserves are broken down during germination, and converted to sugars and transported to cotyledons and axis for dry weight maintenance[13]. Marquez-Millano[14] reported that germination success in *Pinus taeda* declined in proportion to the loss of fatty acid content with seed aging. Edwards et al.[15] reported early germination of seeds produced under elevated $CO_2$, but this was not observed in our study, as seeds from control, elevated $CO_2$, and elevated $O_3$ all had average germination periods of 5–15 days from the day of planting. Germination success rate was significantly higher in elevated $CO_2$ than control and that of elevated $O_3$ was significantly lower than that from ambient. This is similar to results of Hussain et al.[9] and Edwards et al.[15], and implies that seedling recruitment will be enhanced under elevated $CO_2$, but reduced under elevated $O_3$. Additional research needs to be done to find out if the combined elevated

$CO_2$ and $O_3$ treatments will offset one another, as there were no seeds found in this treatment in both the 2004 and 2005 growing seasons.

Seedling vigor was slightly higher in seedlings from elevated $CO_2$ sources and had a decreased mortality rate of 73%. $O_3$ treatment of seeds during their production did not affect seedling survival or vigor. Seedlings from elevated $CO_2$ seeds had higher growth rates compared to those from ambient and elevated $O_3$ sources, but the differences were not significant. These differences might be due to the fact that they had more stored carbohydrate than those from ambient and elevated $O_3$[9]. Even though the seedlings from elevated $CO_2$ were consistently larger than those from $O_3$, the shoot biomass of those from $CO_2$ and $O_3$ were significantly higher than those from control, with $O_3$ being the highest ($CO_2$ - 40% as compared to $O_3$ - 64% increase in shoot biomass). This could probably be due to increased cell wall thickness, lignin, and extractives in the elevated $O_3$ (seedling) as reported by Anderson[16]. It is true that seeds produced in a $CO_2$-enriched environment may have fundamental changes in their viability, chemistry, and germination that may affect reproduction, as predicted by Hussain et al.[9].

### ACKNOWLEDGMENTS

This research was partially supported by the Office of Science (BER), U.S. Department of Energy's Office of Biological and Environmental Research (Grant No. DE-FG02-95ER62125), USDA Forest Service Northern Global Change Program, the USDA Forest Service Central Research Station, Michigan Technological University, the Praxair Foundation, the McIntire-Stennis Program, and Natural Resources Canada-Canadian Forest Service.

The authors are grateful to Janet Pikkarainen for her assistance in editing the manuscript.
